# ﻿Two new species of *Calonectria* (Hypocreales, Nectriaceae) causing *Eucalyptus* leaf blight in Brazil

**DOI:** 10.3897/mycokeys.91.84896

**Published:** 2022-07-29

**Authors:** Enrique I. Sanchez-Gonzalez, Thaissa de Paula Farias Soares, Talyta Galafassi Zarpelon, Edival Angelo Valverde Zauza, Reginaldo Gonçalves Mafia, Maria Alves Ferreira

**Affiliations:** 1 Universidade Federal de Lavras, Departamento de Fitopatologia, Lavras, MG, 37200-900, Brasil Universidade Federal de Lavras Lavras Brazil; 2 Suzano Papel e Celulose S. A. Centro de Tecnologia, Aracruz, ES, 29197-900, Brasil Suzano Papel e Celulose S. A. Centro de Tecnologia Aracruz Brazil

**Keywords:** *Cylindrocladium*, GCPSR, phylogenetic network analysis, phylogeny

## Abstract

In recent decades, commercial *Eucalyptus* plantations have expanded toward the warm and humid regions of northern and northeastern Brazil, where *Calonectria* leaf blight (CLB) has become the primary fungal leaf disease of this crop. CLB can be caused by different *Calonectria* species, and previous studies have indicated that *Calonectria* might have high species diversity in Brazil. During a disease survey conducted in three commercial plantations of *Eucalyptus* in northeastern Brazil, diseased leaves from *Eucalyptus* trees with typical symptoms of CLB were collected, and *Calonectria* fungi were isolated. Based on phylogenetic analyses of six gene regions (*ac*t, *cmdA*, *his3*, *rpb2*, *tef1*, and *tub2*) and morphological characteristics, two new species of *Calonectria* were identified. Five isolates were named as *C.paragominensis***sp. nov.** and four were named as *C.imperata***sp. nov.** The pathogenicity to *Eucalyptus* of both species was confirmed by fulfilling the Koch’s postulates.

## ﻿Introduction

*Calonectria* species are widely distributed around the world and cause diseases in more than 335 plant species, distributed among nearly 100 plant families, including forestry, agricultural and horticultural crops (Crous 2002; Lombard et al. 2010c; Vitale et al. 2013; Lombard et al. 2016; Li et al. 2021). Most reports of *Calonectria* from Brazil are focused on forestry crops, such as *Acacia*, *Eucalyptus*, and *Pinus* trees (Alfenas et al. 2015), and mainly evaluate the epidemiology and disease control of *Calonectria*-associated diseases such as *Calonectria* leaf blight (CLB), damping-off, cutting rot and root rot in commercial plantations and nurseries of *Eucalyptus* (Soares et al. 2018).

Currently, 130 *Calonectria* species have been identified based on DNA phylogenetic analyses and morphological comparisons (Crous et al. 2018, 2019, 2021a, 2021b; Wang et al. 2019; Liu et al. 2020; Mohali and Stewart 2021; Pham et al. 2022). These species are accommodated in eleven species complexes, which are divided into two main phylogenetic groups based on their morphological features: the Prolate Group (*C.brassicae*, *C.candelabrum*, *C.colhounii*, *C.cylindrospora*, *C.gracilipes*, *C.mexicana*, *C.pteridis*, *C.reteaudii* and *C.spathiphylli* species complexes), and the Sphaero-Naviculate Group (*C.kyotensis* and *C.naviculata* species complexes) (Lombard et al. 2016; Liu et al. 2020).

In Brazil, a total of 35 species have been described: eleven species isolated from diseased tissues of *Eucalyptus*, ten species isolated from soil samples of *Eucalyptus* plantations, seven species isolated from different plant species, six species isolated from soil samples of tropical rainforests, and one mycoparasite species (Crous et al. 2018, 2019; Liu et al. 2020); they belong in the species complexes of *C.candelabrum*, *C.brassicae*, *C.cylindrospora*, *C.pteridis*, *C.gracilipes*, and *C.naviculata* (Crous et al. 2018, 2019; Liu et al. 2020). The results from a previous study indicated high species diversity of *Calonectria* in Brazil (Alfenas et al. 2015).

Brazil is one of the main producers of pulp, paper, and wood panels in the world, mainly due to the genus *Eucalyptus*; its hybrids are the most grown trees in the country for these purposes (IBÁ, 2021). In 2020, the total area of *Eucalyptus* plantations was 7.47 million hectares, with an average productivity of 36.8 m^3^/ha per year (IBÁ, 2021). However, in recent decades, commercial *Eucalyptus* plantations have expanded toward the warm and humid regions of northern and northeastern Brazil, where CLB has become the primary fungal leaf disease of this crop (Alfenas et al. 2015). CLB can be caused by different *Calonectria* species, is widely distributed throughout the country, and affects *Eucalyptus* plants most severely from six months to 2–3 years after planting (Graça et al. 2009). This disease starts from spores or microsclerotia present in soil or diseased plant debris on the ground and disseminates to lower branches of the tree canopy; lesions start at the base, apex or margins of leaves and can reach a large area of the leaf blade, resulting in leaf drop and, in some cases, severe defoliation in the basal, middle, and apical thirds of the canopy (Alfenas et al. 2009). The defoliation may decrease timber volume as a result of the reduced photosynthetic area and facilitates weed growth due to the increased entrance of light through the subcanopy, leading to competition for nutrients between *Eucalyptus* and understory plants (Graça et al. 2009; Alfenas et al. 2015).

CLB can be controlled by integrated cultivation and chemical methods as well as by the selection and cultivation of resistant genotypes, which is a much more effective approach (Soares et al. 2018). The demand for new strategies to control this disease requires proper identification of the pathogen species. Additionally, this information may be useful for breeding programs, leading to the development of *Eucalyptus* genotypes resistant to CLB. Recently, during a disease survey conducted in three commercial plantations of *Eucalyptus* in northeastern Brazil, diseased leaves from *Eucalyptus* trees with typical symptoms of CLB were collected, and *Calonectria* fungi were isolated. Thus, the aims of this study were to identify these isolates based on phylogenetic analyses and morphological characteristics and to confirm their pathogenicity to *Eucalyptus*.

## ﻿Materials and methods

### ﻿Sample collection and fungal isolation

In February 2020, during a disease survey conducted in three commercial plantations of *Eucalyptus* on six-month-old to one-year-old trees, diseased leaves with typical symptoms of CLB (small, circular or elongated pale grey to pale brown to dark brown spots, that extend throughout the leaf blade), were observed and collected for fungal isolation and species characterization. On average, 50 diseased leaves were sampled from each *Eucalyptus* genotype, one leaf per tree, depending on the planted areas. The sampled *Eucalyptus* genotypes corresponded to *E.urophylla*, localized in the municipalities of Cidelândia (5°09'24"S, 47°46'26"W) and Itinga do Maranhão (4°34'43"S, 47°29'48"W), in the state of Maranhão, and to the *E.grandis* × *E.brassiana* hybrid genotype, in the microregion of Paragominas (3°10'51"S, 47°18'49"W), in the state of Pará, Brazil.

Samples were stored in paper bags and transported to the Laboratory of Forest Pathology at the Universidade Federal de Lavras. From each leaf, small segments of 1 cm^2^ from the transition section between healthy and diseased tissue were cut and the surface was disinfected by washing with 1% sodium hypochlorite for 1 min, with 70% ethanol for 30 s and with sterilized water three times before culture on 2% malt extract agar (MEA; malt extract 20 g·L^-1^, agar 20 g·L^-1^, yeast extract 2 g·L^-1^, sucrose 5 g·L^-1^) plates at 25 °C. After 48 h of incubation, *Calonectria*-like mycelial plugs, 5 mm in diameter, were transferred to a fresh MEA plate and incubated at 25 °C until the fungus covered the plate completely. Induction of sporulation on MEA plates and single spore cultures was obtained following the procedures described by Alfenas et al. (2013). Each single spore culture was stored and maintained in a metabolically inactive state in dry culture and sterile water following Castellani’s method (Castellani 1939). Holotypes were deposited as herbaria in the Coleção Micológica do Herbário da Universidade de Brasília (UB). Ex-types were deposited as pure cultures in the Coleção de Culturas de Microrganismos do Departamento de Ciência dos Alimentos/UFLA (CCDCA) at Universidade Federal de Lavras (UFLA), Minas Gerais, Brazil. Ex-paratypes were deposited as pure cultures in the Laboratory of Forest Pathology (PFC) at UFLA.

### ﻿DNA extraction, PCR amplification, and sequencing

Total genomic DNA was extracted from fresh mycelia of single spore cultures grown on malt extract broth (MEB; malt extract 20 g·L^-1^, yeast extract 2 g·L^-1^, sucrose 5 g·L^-1^) for ten days at 25 °C in the dark. The protocol described by Lee and Taylor (1990) was followed with slight modifications; by adding 1.5 M NaCl and 2% polyvinylpyrrolidone (MW: 40000) to the lysis buffer; the DNA was precipitated directly with isopropanol without the use of 3 M NaOAc, and the DNA pellet was dried at room temperature overnight. A NanoDrop 1,000 spectrometer (Thermo Fisher Scientific, Waltham, MA, USA) was used to quantify its concentration.

Based on a previous study (Liu et al. 2020), actine (*act*), calmodulin (*cmdA*), histone H3 (*his3*), RNA polymerase II (*rpb2*), translation elongation factor 1-alpha (tef1), and β-tubulin (tub2) genes were used as DNA barcodes due to provide a stable and reliable resolution to distinguish all *Calonectria* species. The primers ACT-512F and ACT-783R (Carbone and Kohn 1999) were used to amplify the *act* gene region; CAL-228F and CAL-2Rd (Carbone and Kohn 1999; Quaedvlieg et al. 2011) for the *cmdA* gene region; CYLH3F and CYLH3R (Crous et al. 2004) for the *his3* gene region; fRpb2-5F and fRpb2-7cR (Liu et al. 1999; Reeb et al. 2004) for the *rpb2* gene region; EF1-728F (Carbone and Kohn 1999) and EF2 (O’Donnell et al. 1998) for the *tef1* gene region and the primer pairs T1 (O’Donnell and Cigelnik 1997) and CYLTUB1R (Crous et al. 2004) for the *tub2* gene region.

The PCRs were carried out in a 25 μL final volume containing molecular biology-grade water (Sigma–Aldrich, St. Louis, MO, USA) 1X PCR buffer (Promega, Madison, WI, USA), 2.5 mM MgCl_2_, 0.2 mM deoxyribonucleotide triphosphate (dNTP) mix (Promega, Madison, WI, USA), 1 U GoTaq Flexi DNA Polymerase (Promega, Madison, WI, USA), 0.2 mM each primer, and 30 ng DNA template. DNA amplifications were conducted in a thermal cycler (5 PRIME G gradient Thermal Cycler, Techne, Staffordshire, UK). The PCR conditions for the *act*, *cmdA*, *his3*, *tef1*, and *tub2* gene regions were as follows: an initial denaturation step at 95 °C for 5 min; then 35 amplification cycles at [94 °C for 30 s; 52 °C for 1 min; 72 °C for 2 min], and a final extension step at 72 °C for 5 min. For the *rpb2* gene region, a touchdown PCR protocol was used: an initial denaturation step at 95 °C for 5 min, then (95 °C for 30 s, 57 °C for 30 s, 72 °C for 90 s) × 10 cycles, (95 °C for 30 s, 57 °C for 45 s, 72 °C for 90 s + 5 s/cycle increase) × 30 cycles, and a final extension step at 72 °C for 10 min.

PCR products were separated by electrophoresis at 120 V for 1 h in a 1.2% agarose gel, stained with Diamond Nucleic Acid Dye (Promega, Madison, WI, USA), and visualized using an ultraviolet light transilluminator. Successful PCR products were purified and sequenced in both directions using the same primer pairs used for amplification by Macrogen Inc. (Macrogen, Seoul, Korea). Raw sequences from each gene region were edited, consensus sequences were generated using SeqAssem software ver. 07/2008 (Hepperle 2004), and the sequences generated in this study were deposited in the NCBI/GenBank database (http://www.ncbi.nlm.nih.gov).

### ﻿Phylogenetic analyses

The generated sequences were aligned with other sequences of closely related *Calonectria* spp. obtained from GenBank (Table 1), using the online interface of MAFFT v. 7.0 (Katoh et al. 2019, http://mafft.cbrc.jp/alignment/server) with the alignment strategy FFT-NS-i (Slow; interactive refinement method). Alignments were manually corrected using MEGA7 (Kumar et al. 2016).

**Table 1.** *Calonectria* species and GenBank accession numbers of DNA sequences used in this study.

Species complex	Species	Isolate representing the species^‡,§^	Other isolate numbers	Host/ Substrate	Country	Genbank accession numbers					
						*act* ^|^	*cmdA*	*his3*	*rpb2*	*tef1*	*tub2*
*Calonectriaspathiphylli* species complex	*C.densa*	**CMW 31182**		Soil	Ecuador	GQ280525	GQ267444	GQ267281	N/A	GQ267352	GQ267232
		CMW 31184		Soil	Ecuador	GQ280523	GQ267442	GQ267279	N/A	GQ267350	GQ267230
		CMW 31185		Soil	Ecuador	GQ280524	GQ267443	GQ267280	N/A	GQ267351	GQ267231
	*C.humicola*	**CMW 31183**		Soil	Ecuador	GQ280526	GQ267445	GQ267282	N/A	GQ267353	GQ267233
		CMW 31186		Soil	Ecuador	GQ280527	GQ267446	GQ267283	N/A	GQ267354	GQ267234
		CMW 31187		Soil	Ecuador	GQ280528	GQ267447	GQ267284	N/A	GQ267355	GQ267235
	***C.paragominensis* sp. nov.** ^†^	**CCDCA 1164**8		*E.grandis* × *E.brassiana*	Brazil	**ON009346**	**OM974325**	**OM974334**	**OM974343**	**OM974352**	**OM974361**
		PFC2		*E.grandis* × *E.brassiana*	Brazil	**ON009347**	**OM974326**	**OM974335**	**OM974344**	**OM974353**	**OM974362**
		PFC3		*E.grandis* × *E.brassiana*	Brazil	**ON009348**	**OM974327**	**OM974336**	**OM974345**	**OM974354**	**OM974363**
		PFC4		*E.grandis* × *E.brassiana*	Brazil	**ON009349**	**OM974328**	**OM974337**	**OM974346**	**OM974355**	**OM974364**
		PFC5		*E.grandis* × *E.brassiana*	Brazil	**ON009350**	**OM974329**	**OM974338**	**OM974347**	**OM974356**	**OM974365**
	*C.pseudospathiphylli*	**CBS 109165**	CPC 1623	Soil	Ecuador	GQ280493	GQ267412	AF348241	KY653435	FJ918562	AF348225
		CPC 1641		Soil	Ecuador	N/A	N/A	AF348233	N/A	N/A	AF348217
	*C.spathiphylli*	**CBS 114540**	ATCC44730, CSF11330	*Spathiphyllum* sp.	USA	GQ280505	GQ267424	AF348230	MT412666	GQ267330	AF348214
		CBS 116168	CSF 11401	*Spathiphyllum* sp.	Switzerland	GQ280506	GQ267425	FJ918530	MT412667	FJ918561	FJ918512
*Calonectriacandelabrum* species complex	*C.brasiliana*	**CBS 111484**	CSF 11249	Soil	Brazil	MT334968	MT335198	MT335438	MT412502	MT412729	MT412951
		CBS 111485	CSF 11250	Soil	Brazil	MT334969	MT335199	MT335439	MT412503	MT412730	MT412952
	*C.brassiana*	**CBS 134855**		Soil	Brazil	N/A	KM396056	KM396139	N/A	KM395882	KM395969
		CBS 134856		Soil	Brazil	N/A	KM396057	KM396140	N/A	KM395883	KM395970
	*C.brevistipitata*	**CBS 115671**	CSF 11288	Soil	Mexico	MT334973	MT335203	MT335443	MT412507	MT412734	MT412956
		CBS 110928	CSF 11235	Soil	Mexico	MT334974	MT335204	MT335444	MT412508	MT412735	MT412957
	*C.candelabrum*	**CMW 31000**	CSF 11404	*Eucalyptus* sp.	Brazil	MT334977	MT335207	MT335447	MT412511	MT412738	MT412959
		CMW 31001	CSF 11405	*Eucalyptus* sp.	Brazil	MT334978	MT335208	MT335448	MT412512	MT412739	MT412960
	*C.colombiana*	**CBS 115127**		Soil	Colombia	GQ280538	GQ267455	FJ972442	N/A	FJ972492	FJ972423
		CBS 115638		Soil	Colombia	GQ280539	GQ267456	FJ972441	N/A	FJ972491	FJ972422
	*C.eucalypticola*	**CBS 134847**		*Eucalyptus* sp.	Brazil	N/A	KM396051	KM396134	N/A	KM395877	KM395964
		CBS 134846		*Eucalyptus* sp.	Brazil	N/A	KM396050	KM396133	N/A	KM395876	KM395963
	*C.fragariae*	**CBS 133607**		Fragaria×ananassa	Brazil	N/A	KM998966	KM998964	N/A	KM998963	KM998965
		LPF141.1		Fragaria×ananassa	Brazil	N/A	KX500191	KX500194	N/A	KX500197	KX500195
	*C.glaebicola*	**CBS 134852**		Soil	Brazil	N/A	KM396053	KM396136	N/A	KM395879	KM395966
		CBS 134853		*Eucalyptus* sp.	Brazil	N/A	KM396054	KM396137	N/A	KM395880	KM395967
	*C.hemileiae*	**COAD 2544**		*Hemileiavastatrix*	Brazil	N/A	MK037392	MK006026	N/A	MK006027	MK037391
*Calonectriacandelabrum* species complex	***C.imperata* sp. nov.** ^†^	**CCDCA 11649**		*E.urophylla*	Brazil	**ON009351**	**OM974330**	**OM974339**	**OM974348**	**OM974357**	**OM974366**
		PFC7		*E.urophylla*	Brazil	**ON009352**	**OM974331**	**OM974340**	**OM974349**	**OM974358**	**OM974367**
		PFC8		*E.urophylla*	Brazil	**ON009353**	**OM974332**	**OM974341**	**OM974350**	**OM974359**	**OM974368**
		PFC9		*E.urophylla*	Brazil	**ON009354**	**OM974333**	**OM974342**	**OM974351**	**OM974360**	**OM974369**
	*C.matogrossensis*	**GFP 006**		*E.urophylla*	Brazil	N/A	MH837653	MH837648	N/A	MH837659	MH837664
		GFP 018		*E.urophylla*	Brazil	N/A	MH837657	MH837652	N/A	MH837663	MH837668
	*C.metrosideri*	**CBS 133603**		*Metrosiderospolymorpha*	Brazil	N/A	KC294304	KC294307	N/A	KC294310	KC294313
		CBS 133604	CSF 11309	*Metrosiderospolymorpha*	Brazil	MT335056	MT335288	MT335528	MT412585	MT412819	MT413033
	*C.nemoricola*	**CBS 134837**		Soil	Brazil	N/A	KM396066	KM396149	N/A	KM395892	KM395979
		CBS 134838		Soil	Brazil	N/A	KM396067	KM396150	N/A	KM395893	KM395980
	*C.pauciramosa*	**CBS 138824**	CSF 16461	Soil	South Africa	MT335093	MT335325	MT335565	MT412618	MT412856	MT413068
		CMW 31474	CSF 11422	*E.urophylla* × *E.grandis*	China	MT335104	MT335336	MT335576	MT412629	MT412867	MT413079
	*C.piauiensis*	**CBS 134850**		Soil	Brazil	N/A	KM396060	KM396143	N/A	KM395886	KM395973
		CBS 134851		Soil	Brazil	N/A	KM396061	KM396144	N/A	KM395887	KM395974
	*C.pseudometrosideri*	**CBS 134845**		Soil	Brazil	N/A	KM395995	KM396083	N/A	KM395821	KM395909
		CBS 134843		*Metrosiderospolymorpha*	Brazil	N/A	KM395993	KM396081	N/A	KM395819	KM395907
	*C.pseudospathulata*	**CBS 134841**		Soil	Brazil	N/A	KM396070	KM396153	N/A	KM395896	KM395983
		CBS 134840		Soil	Brazil	N/A	KM396069	KM396152	N/A	KM395895	KM395982
	*C.putriramosa*	**CBS 111449**	CSF 11246	*Eucalyptus* cutting	Brazil	MT335129	MT335364	MT335604	MT412657	MT412895	MT413105
		CBS 111470	CSF 11247	Soil	Brazil	MT335130	MT335365	MT335605	MT412658	MT412896	MT413106
	*C.silvicola*	**CBS 135237**	LPF081	Soil	Brazil	N/A	KM396065	KM396148	N/A	KM395891	KM395978
		CBS 134836		Soil	Brazil	N/A	KM396062	KM396145	N/A	KM395888	KM395975
	*C.spathulata*	**CMW 16744**	CSF 11331	*E.viminalis*	Brazil	MT335139	MT335376	MT335616	MT412668	MT412907	MT413117
		CBS 112513	CSF 11259	*Eucalyptus* sp.	Colombia	MT335140	MT335377	MT335617	MT412669	MT412908	MT413118
	*C.venezuelana*	**CBS 111052**	CSF 11238	Soil	Venezuela	MT335155	MT335394	MT335634	MT412685	MT412925	MT413132
*Calonectriagracilipes* species complex	*C.gracilipes*	**CBS 115674**	CSF 11289	Soil	Colombia	MT335022	MT335252	MT335492	MT412554	MT412783	MT413001
		CBS 111141	CSF11239	Soil	Colombia	MT335023	MT335253	MT335493	MT412555	MT412784	MT413002

† New *Calonectria* species reported in the present study. ‡ Ex-type isolates of the *Calonectria* species are marked in bold. § ATCC: American Type Culture Collection, Virginia, USA; CBS: Westerdijk Fungal Biodiversity Institute, Utrecht, The Netherlands; CCDCA: Coleção de Culturas de Microrganismos do Departamento de Ciência dos Alimentos/UFLA, Lavras, Brazil; CMW: Culture collection of the Forestry and Agricultural Biotechnology Institute (FABI), University of Pretoria, Pretoria, South Africa; COAD: Coleção Octávio de Almeida Drumond, Universidade Federal de Viçosa, Viçosa, Brazil; CPC: Pedro Crous working collection housed at Westerdijk Fungal Biodiversity Institute; CSF: Culture Collection located at China Eucalypt Research Centre (CERC), Chinese Academy of Forestry, ZhanJiang, GuangDong Province, China; GFP: Universidade Federal de Brasilia, Brasilia, Brazil; LPF: Laboratorio de Patologia Florestal, Universidade Federal de Viçosa, Viçosa, Brazil; PFC: Laboratorio de Patologia Florestal, Universidade Federal de Lavras, Lavras, Brazil. | *act*: actin; *cmdA*: calmodulin; *his3*: histone H3; *rpb2*: the second largest subunit of RNA polymerase; *tef1*: translation elongation factor 1-alpha; *tub2*: β-tubulin. GenBank accession number obtained in this study are marked in bold.

The partition homogeneity test (PHT) described by Farris et al. (1995) was conducted to determine if data for six genes could be combined using PAUP 4.0b10 (Swofford 2003). To determine the phylogenetic relationships among species, phylogenetic analyses based on maximum parsimony (MP), maximum likelihood (ML), and bayesian inference (BI) were conducted on the individual gene regions and their concatenated dataset, depending on the sequence availability.

Maximum parsimony analysis was performed using PAUP 4.0b10 (Swofford 2003), with phylogenetic relationships estimated by heuristic searches, random stepwise addition sequences, and tree bisection and reconnection (TBR) branch swapping. Gaps were treated as missing data, and all characters were unordered and weighted equally. The measures calculated for parsimony included the tree length (TL), consistency index (CI), homoplasy index (HI), retention index (RI), and rescaled consistency index (RC). Statistical support for branch nodes was assessed with 1,000 bootstrap replicates.

The best evolutionary model of nucleotide substitution for each gene region was selected according to the Akaike Information Criterion (AIC) using MODELTEST v. 3.4 (Posada and Crandall 1998) for ML analyses and MRMODELTEST v. 2 (Nylander 2004) for BI analyses.

ML analyses for individual gene regions were performed using PAUP 4.0b10 (Swofford 2003). The ML models used were K80 + G (*act*), TVM + G (*cmdA*), TrN + G (*his3*), SYM + G (*rpb2*), TVM + I + G (*tef1*) and HKY + I (*tub2*). Statistical support for branch nodes was assessed with 1,000 bootstrap replicates. A partitioned ML analysis was performed using IQ-TREE (Nguyen et al. 2015) as implemented in the IQ-TREE web server (http://iqtree.cibiv.univie.ac.at, Trifinopoulos et al. 2016) by using partition models (Chernomor et al. 2016). Branch support values were evaluated based on 10,000 replicates for ultrafast bootstrapping (UFBoot2) (Hoang et al. 2018).

Individual and partitioned BI analyses were performed using MRBAYES v.3.2.7a (Ronquist et al. 2012) on XSEDE at the CIPRES Science Gateway v.3.3 (http://www.phylo.org/). The BI models used were K80 + G (*act*), GTR + G (*cmdA* and *his3*), SYM + G (*rpb2*), GTR + I + G (*tef1*) and HKY + I (*tub2*). A Markov Chain Monte Carlo (MCMC) algorithm was employed, and two independent runs of four MCMC chains (three hot and one cold) were run in parallel simultaneously starting from random trees for 10^7^ generations (individual gene regions) and 30^7^ generations (concatenated dataset), sampling trees every 1,000 generations. The distribution of log-likelihood scores was examined with TRACER v.1.5 (Rambaut and Drummond 2007) to determine the whether the stationary phase of each search was reached and whether chains had achieved convergence. The convergence of the chains was also assessed by the convergent diagnostics of the effective sampling site (ESS), the potential scale reduction factor (PSRF), and the average standard deviation of split frequencies (ASDSF) (Ronquist et al. 2019). The first 25% of saved trees were discarded as the “burn-in” phase, and posterior probabilities (PP) were computed using the remaining trees. Trees were visualized in FIGTREE v. 1.4.4 (Rambaut 2009) and edited in INKSCAPE v. 1.0 (https://inkscape.org).

### ﻿Pairwise homoplasy index (PHI) test and phylogenetic network analysis

Phylogenetically closely related species were analyzed using the Genealogical Concordance Phylogenetic Species Recognition (GCPSR) model (as described by Taylor et al. 2000) by performing a pairwise homoplasy index (Φw) test (PHI) (Bruen et al. 2006). The PHI test was performed in SPLIT TREE4 v.4.16.1. (https://uni-tuebingen.de) (Huson and Bryant 2006) to determine the recombination level within phylogenetically closely related species. Only the gene regions that were available for all compared individuals were used. Gaps’ sites were excluded. Significant recombination was considered at a PHI index below 0.05 (Φw < 0.05). The relationships between closely related taxa were visualized by constructing a phylogenetic network from the concatenated datasets using the LogDet transformation and the NeighborNet method; the resultant networks were displayed with the EqualAngle algorithm (Dress and Huson 2004). Bootstrap analysis was then conducted with 1,000 replicates.

### ﻿Mating type and sexual compatibility test

The mating-type idiomorph of each *Calonectria* species isolate was determined through PCR by using the primer pairs Cal_MAT111_F/Cal_MAT111_R and Cal_MAT121_F/Cal_MAT121_R, which amplify the MAT1-1-1 and MAT1-2-1 genes using the protocol described by Li et al. (2020). Additionally, sexual compatibility tests were performed for all the single-spore isolates of both species on minimal salt agar (MSA; Guerber and Correll 2001) by crossing them in all possible combinations, following the procedure described by Lombard et al. (2010a). The plates were stacked in plastic bags and incubated at 25 °C for 12 weeks.

### ﻿Morphology

Morphological characterization of representative isolates of each *Calonectria* species identified by phylogenetic analyses was performed as described by Liu and Chen (2017). Optimal growth temperatures were determined by incubating the representative isolate at temperatures ranging from 5 °C to 30 °C at 5 °C intervals in the dark on MEA plates (three replicates per isolate were used). Colonial characteristics (diameter, color, and texture of colonies) were determined by inoculating the isolates on MEA plates at 25 °C in the dark after seven days of incubation.

### ﻿Pathogenicity tests

One representative isolate of each *Calonectria* species was selected for inoculation. Healthy leaves of three short cut branches from an approximately eleven-month-old *Eucalyptus* plants were inoculated with suspensions of 1 × 10^4^ conidia·mL^-1^ obtained from single spore cultures. The conidia suspensions for each isolate were prepared using the method described by Graça et al. (2009). *Calonectriaparagominensis* was inoculated on *E.grandis* × *E.brassiana* hybrid genotype and *C.imperata* on *E.urophylla* genotype. The inoculation consisted of spraying the conidia suspension until the suspension run off the leaves. Sterile water was sprayed onto healthy leaves as the negative control. The branches with inoculated leaves were covered with plastic bags to maintain high humidity and kept at 25 °C under a photoperiod of 12 h for 72 h. After that time, the plastic bags were removed, and necrotic symptoms were observed.

## ﻿Results

### ﻿Fungal isolates

A total of 34 isolates with the typical morphology of *Calonectria* species were obtained from infected leaves of the *Eucalyptus* genotypes sampled. Based on preliminary phylogenetic analyses of the *tef1* and *tub2* gene regions (data not shown), nine isolates were selected for further studies (Table 1).

### ﻿Phylogenetic analyses

Sequences from 50 isolates corresponding to 25 *Calonectria* species closely related to the isolates obtained in this study were downloaded from GenBank (Table 1). For the nine isolates selected in this study, five resided in the *Calonectriaspathiphylli* species complex (CSSC), and four resided in the *Calonectriacandelabrum* species complex (CCSC). Both *Calonectria* complexes belong to the Prolate Group, whose species are characterized by their clavate to pyriform to ellipsoidal vesicles (Liu et al. 2020). Therefore, both complexes were combined into a single sequence dataset for phylogenetic analyses, including two strains of *Calonectriagracilipes* as the outgroup taxa.

Alignments for each gene region and the concatenated dataset were as follows: *act* (36 isolates, 267 characters), *cmdA* (58 isolates, 485 characters), *his3* (59 isolates, 439 characters), *rpb2* (28 isolates, 863 characters), *tef1* (58 isolates, 496 characters), *tub2* (59 isolates, 511 characters) and concatenated (59 isolates, 3061 characters). The PHT generated a *p* value of 0.01 for the concatenated dataset, suggesting some incongruence in the datasets for the six regions and the accuracy of the combined data could have suffered relative to the individual partitions (Cunningham 1997). Although the *p* value was low, the different gene regions were combined because the significance threshold of 0.05 may be too conservative and it has been shown that combining incongruent datasets improves phylogenetic accuracy (Sullivan 1996; Cunningham 1997); moreover, this approach was followed by several previous studies (Lombard et al. 2016; Pham et al. 2019; Liu et al. 2020, 2021).

**Figure 1.**
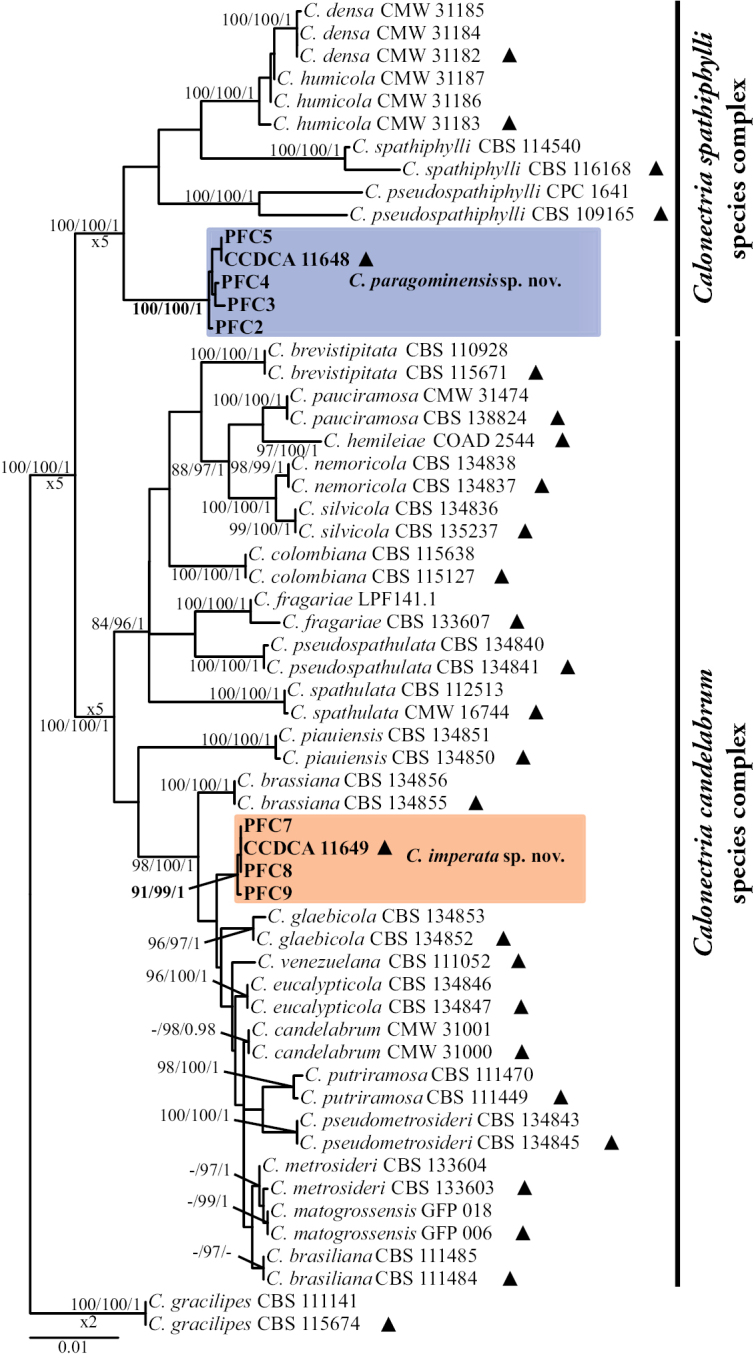
Phylogenetic tree based on maximum likelihood analysis of concatenated *act*, *cmdA*, *his3*, *rpb2*, *tef1* and *tub2* gene regions. Bootstrap support values ≥ 80% for maximum parsimony (MP), Ultrafast bootstrap support values ≥ 95% for maximum likelihood (ML), and posterior probability (PP) values ≥ 0.95 from BI analyses are presented at the nodes (MP/ML/PP). Bootstrap values below 80% (MP), 95% (ML) and posterior probabilities below 0.80 are marked with “-”. Ex-type isolates are indicated by “▲”, isolates highlighted in bold were sequenced in this study, and novel species are in blue and orange. *C.gracilipes* was used as outgroup. The scale bar indicates the number of nucleotide substitutions per site.

Tree topologies derived from the MP, ML, and BI analyses of the individual gene regions were similar overall, but the relative positions of some *Calonectria* species slightly differed. Moreover, the concatenated dataset formed well-supported lineages in the MP, ML, and BI analyses. Only the ML trees are presented in this study (Fig. 1, Suppl. material 1: Figs S1–S6). The concatenated dataset had 466 parsimony-informative characters, 67 parsimony-uninformative characters, and 2,528 constant characters. Analysis of the 466 parsimony-informative characters yielded 2 equally parsimonious trees, with TL = 862, CI = 0.7042, HI = 0.2958, RI = 0.9192, RC = 0.6472. For the partitioned BI analysis, the convergence of the chains was confirmed by an ESS > 200, a PSRF approaching 1, and an ASDSF equal to 0.000793. The aligned sequences were deposited in TreeBASE (http://treebase.org; No. 29573).

Phylogenetic analyses of the six individual gene regions showed that the five isolates from the CSSC were clustered in an independent clade (Suppl. material 1: Figs S1–S6). Based on the concatenated dataset of the six genes, the five isolates formed a new, strongly defined phylogenetic clade that was distinct from the other *Calonectria* species of the CSSC and was supported by high bootstrap values (MP = 100%, ML = 100%) and high values of posterior probability (1.0) (Fig. 1). A total of 41 fixed unique single nucleotide polymorphisms (SNPs) were identified in the new phylogenetic clade of the five isolates in comparison with their phylogenetically closely related *Calonectria* species in the six-gene concatenated dataset (Table 2). The results of these phylogenetic and SNP analyses indicate that the five isolates in the CSSC represent a distinct, undescribed species, which we named *C.paragominesis*.

**Table 2.** Single nucleotide polymorphisms unique to *C.paragominensis* in comparison with their phylogenetically closely related species in the six gene regions.

Species	*act* ^†^										*cmdA*										
	188^‡^	189	190	191	192	193	194	195	196	197	142	144	170	185	217	270	437	444	455	483	
*C.paragominensis*CCDCA 11648	a	g	a	a	a	a	a	g	a	a	t	a	c	c	t	c	a	a	g	a	
*C.densa*CMW 31182	t	-	-	-	-	-	-	-	-	-	a	c	t	t	c	t	g	g	a	c	
*C.humicola*CMW 31183	t	-	-	-	-	-	-	-	-	-	a	c	t	t	c	t	g	g	a	c	
*C.pseudospathiphylli*CBS 109165	t	-	-	-	-	-	-	-	-	-	a	c	t	t	c	t	g	g	a	c	
*C.spathiphylli*CBS 114540	t	-	-	-	-	-	-	-	-	-	a	c	t	t	c	t	g	g	a	c	
**Species**	***his3***									***rpb2***						***tef1***					***tub2***
	**6**	**43**	**47**	**52**	**53**	**54**	**247**	**259**	**274**	**81**	**141**	**315**	**474**	**630**	**735**	**32**	**123**	**208**	**434**	**459**	**15**
*C.paragominensis*CCDCA 11648	c	t	t	-	-	-	a	g	a	t	c	t	t	a	g	c	t	g	g	c	a
*C.densa*CMW 31182	t	a	c	c	t	c	t	a	g							-	-	a	t	t	t
*C.humicola*CMW 31183	t	a	c	c	t	c	t	a	g							-	-	a	t	t	t
*C.pseudospathiphylli*CBS 109165	-	a	c	c	a	c	c	a	g	c	t	c	c	g	a	-	-	a	a	t	t
*C.spathiphylli*CBS 114540	-	a	c	c	c	c	c	a	g	c	t	c	c	g	a	-	-	a	t	t	t

† Only polymorphic nucleotides occurring in all the isolates are shown, not alleles that partially occur in individuals per phylogenetic group. ‡ Numerical positions of the nucleotides in the DNA sequence alignments.

Phylogenetic analyses of the individual gene regions of *act*, *cmdA*, *his3*, *rpb2*, and *tub2* showed that the four isolates that resided in the CCSC were clustered in an independent clade (Suppl. material 1: Figs S1–S4, S6). However, the phylogenetic tree based on *tef1* showed that three of those isolates formed an independent clade, while one isolate was closely related to *C.metrosideri*, *C.pseudometrosideri*, and *C.candelabrum* (Suppl. material 1: Fig. S5). Based on the concatenated dataset of the six genes, the four isolates formed a new, strongly defined phylogenetic clade that was distinct from other *Calonectria* species in the CSSC and was supported by high bootstrap values (MP = 91%, ML = 99%) and high values of posterior probability (1.0) (Fig. 1). The four isolates of the new phylogenetic clade were distinguished from their phylogenetically closely related *Calonectria* species using SNP analyses for the six-gene concatenated dataset, by presenting eight unique SNPs from a total of 78 SNPs (Table 3). The results of these phylogenetic and SNP analyses indicate that the four isolates in the CCSC represent a distinct, undescribed species, which we named *C.imperata*.

**Table 3.** Single nucleotide polymorphisms found in *Calonectriaimperata* and its phylogenetically closely related species in the six gene regions.

Species	*act* ^†^	*cmdA*												*his3*						
	57^‡^	62	71	121	171	187	210	319	376	403	405	418	444	8	44	46	53	56	60	66
*C.imperata*CCDCA 11649	**c**	a	c	c	g	g	c	c	t	**t**	c	**t**	t	c	c	t	c	c	g	a
*C.brassiana*CBS 134855		c	g	t	g	g	c	c	c	c	c	a	t	t	c	t	c	c	g	a
*C.glaebicola*CBS 134852		a	c	c	g	g	c	g	t	c	c	a	t	c	t	c	c	c	g	a
*C.piauiensis*CBS 134850		a	g	c	c	a	c	c	c	c	c	a	c	c	c	-	a	c	a	c
*C.venezuelana*CBS 111052	t	a	c	c	g	g	a	g	t	c	t	a	t	c	c	t	c	t	g	a
**Species**	***his3***																			
	**93**	**99**	**105**	**114**	**156**	**189**	**234**	**235**	**238**	**244**	**245**	**250**	**251**	**252**	**254**	**255**	**257**	**262**	**275**	**276**
*C.imperata*CCDCA 11649	c	c	c	a	t	t	t	t	c	c	a	c	c	a	g	c	a	a	t	g
*C.brassiana*CBS 134855	c	c	c	t	t	t	t	t	c	c	a	c	c	a	g	c	a	a	t	g
*C.glaebicola*CBS 134852	c	c	c	a	t	t	t	t	c	a	a	c	c	a	g	c	a	a	t	g
*C.piauiensis*CBS 134850	t	t	c	a	c	t	c	g	t	g	g	t	a	g	a	t	g	g	c	a
*C.venezuelana*CBS 111052	c	c	a	a	t	c	t	t	c	c	a	c	c	a	g	c	a	a	t	g
**Species**	***his3***							***rpb2***					***tef1***							
	**277**	**278**	**333**	**336**	**351**	**405**	**420**	**105**	**603**	**624**	**693**	**840**	**47**	**81**	**110**	**112**	**135**	**220**	**239**	**357**
*C.imperata*CCDCA 11649	c	t	t	c	g	c	t	**g**	**a**	**c**	**t**	**a**	c	g	a	t	t	c	c	c
*C.brassiana*CBS 134855	c	t	t	c	g	t	t						c	g	t	t	t	c	c	c
*C.glaebicola*CBS 134852	c	t	t	c	a	c	t						c	a	a	t	t	c	c	c
*C.piauiensis*CBS 134850	t	t	c	t	g	c	g						t	g	a	a	c	a	t	c
*C.venezuelana*CBS 111052	c	c	t	c	g	c	t	t	g	t	c	t	c	g	a	t	t	c	c	t
**Species**	***tef1a***					***tub2***														
	**417**	**421**	**422**	**425**	**453**	**50**	**99**	**120**	**132**	**174**	**175**	**188**	**191**	**220**	**377**	**398**	**408**	**409**		
*C.imperata*CCDCA 11649	c	c	c	a	a	c	a	g	t	c	c	g	c	t	t	t	-	-		
*C.brassiana*CBS 134855	c	t	t	a	a	c	a	g	t	c	c	g	c	t	t	t	a	c		
*C.glaebicola*CBS 134852	c	t	t	a	a	c	a	g	t	c	c	g	c	t	t	t	a	c		
*C.piauiensis*CBS 134850	t	c	c	a	a	g	g	a	c	t	t	a	a	c	c	g	a	c		
*C.venezuelana*CBS 111052	c	c	c	c	g	c	a	g	t	c	c	g	c	t	t	t	a	c		

† Only polymorphic nucleotides occurring in all the isolates are shown, not alleles that partially occur in individuals per phylogenetic group. ‡ Numerical positions of the nucleotides in the DNA sequence alignments.

### ﻿Species delimitation by GCPSR analysis

**Figure 2.**
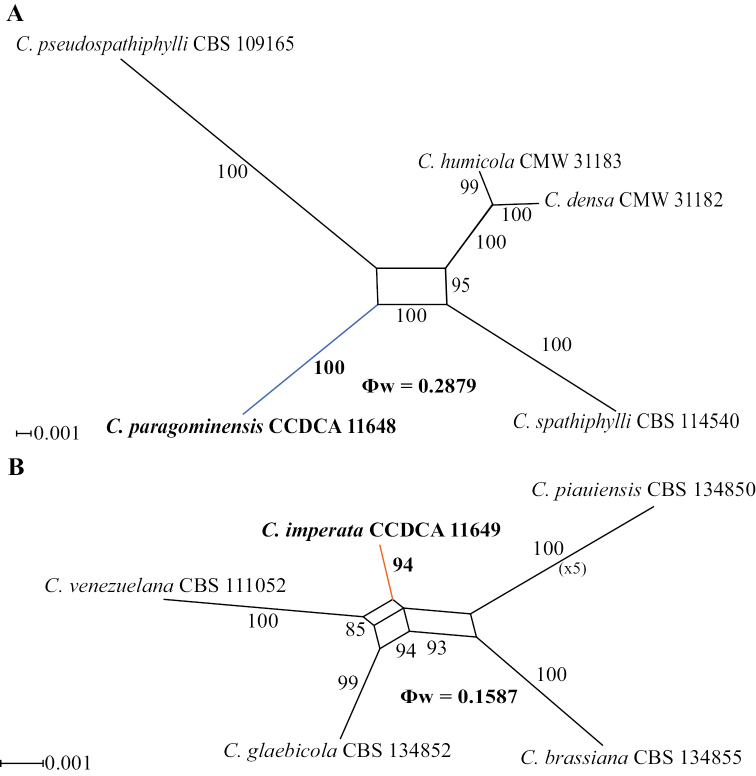
Results of the pairwise homoplasy index (PHI) test for *C.paragominensis* and *C.imperata*. Phylogenetic networks constructed using the LogDet transformation and the NeighborNet method and displayed with the EqualAngle algorithm. Bootstrap support values > 80% are shown. Φw < 0.05 indicate significant recombination. New species described in this study are highlighted in bold, with blue (**A**) and orange (**B**) lines.

A PHI test using a five-locus concatenated dataset (*act*, *cmdA*, *his3*, *tef1*, *tub2*) was performed to determine the recombination level among *C.paragominensis* and its phylogenetically closely related species, *C.densa*, *C.humicola*, *C.spathiphylli* and *C.pseudospathiphylli*. A value of Φw = 0.2879 revealed no significant genetic recombination events, and this relationship was supported with a high bootstrap value (100%) in the phylogenetic network analysis, indicating that they are different species (Fig. 2A).

A PHI test using a four-locus concatenated dataset (*cmdA*, *his3*, *tef1*, *tub2*) was performed to determine the recombination level among *C.imperata* and its phylogenetically closely related species, *C.brassiana*, *C.glabeicola*, *C.piauiensis*, and *C.venezuelana*. A value of Φw = 0.1587 revealed no significant genetic recombination events, and this relationship was supported with a high bootstrap value (94%) in the phylogenetic network analysis, indicating that they are different species (Fig. 2B).

### ﻿Mating-type and sexual compatibility test

MAT1-1-1 and MAT1-2-1 genes were amplified in all isolates of each identified species, indicating that they are putatively homothallic. However, after a twelve-week mating test on MSA, all isolates failed to yield sexual structures, indicating that they have lost the ability to be self-fertile or have retained the ability to favor outcrossing rather than selfing.

### ﻿Taxonomy

Based on phylogenetic analyses, GCPSR, and network analyses, the nine isolates presented two strongly defined phylogenetic clades in both the *Calonectriaspathiphylli* species complex and the *Calonectriacandelabrum* species complex. Morphological differences, especially in the macroconidia and stipe dimensions, were observed between each phylogenetic clade and its phylogenetically closely related species (Table 4). Thus, the fungi isolated in this study represent two new species of *Calonectria* and are described as follows:

**Table 4.** Morphological characteristics of two new *Calonectria* species and their phylogenetically closely related species.

Species complex	Species	Conidiogenus apparatus		Stipe		Macroconidia			Vesicle		Reference
		Size (L × W)^†,‡,§^	Branches	Size (L × W)^†,‡,§^	Extension (L × W)^†,‡,§^	Size (L × W)^†,‡,§,|^	Average (L × W)^†,‡,§^	Septa	Diameter^†,§^	Shape	
***Calonectriaspathiphylli* species complex**	***C.paragominensis***	40–113 × 45–129	(–4)	112–281 × 2–4	123–295 × 1.5–3	(47–)56–66(–71) × (4–)4.8–5.9(–7)	61 × 5	1(–3)	8–12	globoid to sphaeropedunculate	This study
	*C.densa*	49–78 × 63–123	(–4)	54–90 × 6–10	149–192 × 5–6	(47–)50–58(–62) × (5–)6	54 × 6	1	10–12	ovoid to ellipsoid to sphaeropedunculate	Lombard et al. 2010b
	*C.humicola*	43–71 × 42–49	3	44–90 × 6–8	126–157 × 4–5	(45–)48–54(–56) × (4–)5	51 × 5	1	10–12	globoid to ovoid to sphaeropedunculate	Lombard et al. 2010b
	*C.pseudospathiphylli*	70–100 × 25–70	4	100–350 × 5–6	100–250 × 2.5–3.5	(40–)47–55(–60) × 4–5	52 × 4	1(–3)	8–12	sphaeropedunculate to ellipsoid	Kang et al. 2001; Crous 2002
	*C.spathiphylli*	60–150 × 40–90	4	120–150 × 6–8	170–260 × 3–4	(45–)65–80(–120) × (5–)6(–7)^¶^	70 × 6	1(–3)	8–15	globoid or ellipsoid to obpyriform	Crous 2002
***Calonectriacandelabrum* species complex**	***C.imperata***	50–127 × 41–110	(–3)	135–227 × 2–4	151–254 × 1.5–3	(38–)43–49(–52) × (2–)2.7–3.2(–4)	46 × 3	(–1)	3–6	ellipsoid to narrowly obpyriform	This study
	*C.piauiensis*	20–60 × 35–80	2	50–110 × 4–6	95–130 × 2–3	(38–)47–52(–60) × 3–5	49 × 4.5	1	3–7	ellipsoid to narrowly obpyriform	Alfenas et al. 2015
	*C.brassiana*	50–80 × 50–135	3	55–155 × 5–8	90–172 × 2–3	(35–)50–56(–65) × 3–5	53 × 4	1	3–7	ellipsoid to narrowly obpyriform	Alfenas et al. 2015
	*C.glaebicola*	27–45 × 25–40	2	50–130 × 5–7	100–165 × 2–4	(45–)50–52(–55) × 3–5	50 × 4	1	3–5	ellipsoid to narrowly obpyriform	Alfenas et al. 2015
	*C.venezuelana*	25–65 × 25–60	3	35–100 × 4–8	85–190 × 3–6	(48–)54–62(–65) × (4–)4.5–5.5(–7)	58 × 5	1	5–9	fusiform to ovoid to ellipsoid	Lombard et al. 2016

† All measurements are in μm. ‡ L × W = length × width. § Minimum–maximum. | Measurements are presented in the format [(minimum–) (average – standard deviation) – (average + standard deviation) (–maximum)]. ¶ Measurements are presented in the format [(minimum–) (average) (–maximum)].

#### ﻿ Calonectria paragominensis

Taxon classificationFungiHypocrealesNectriaceae

E.I.Sanchez, T.P.F.Soares & M.A.Ferreira sp. nov.

F9C48BD4-5C29-5A44-9515-0AD7F88CEAA8

843460

Fig. 3

##### Etymology.

The term “*paragominensis*” refers to the microregion of Paragominas, Brazil, which is the place where the fungus was collected.

##### Diagnosis.

*Calonectriaparagominensis* differs from the phylogenetically closely related species *C.densa*, *C.humicola*, *C.spathiphylli* and *C.pseudospathiphylli* with respect to its macroconidia dimensions.

##### Type.

**Brazil**,• Pará state, Paragominas microregion; 3°10'51"S, 47°18'49"W; From infected leaves of *E.grandis* × *E.brassiana*; 20 Feb. 2020; M.A. Ferreira; **holotype**: UB24349, **ex-type**: CCDCA 11648 = PFC1. GenBank: *act* = ON009346; *cmdA* = OM974325; *his3* = OM974334; *rpb2* = OM974343; *tef1* = OM974352; *tub2* = OM974361.

##### Description.

Sexual morph unknown. Macroconidiophores consisted of a stipe, a suite of penicillate arrangements of fertile branches, a stipe extension, and a terminal vesicle; stipe septate, hyaline, smooth, (112–)135–207(–281) × (2–)2.6–3.5(–4) μm; stipe extension septate, straight to flexuous, (123–)147–220(–295) μm long, (1.5–)1.9–2.4(–3) μm wide at the apical septum, terminating in a globose to sphaeropedunculate vesicle, (8–)8.5–10.5(–12) μm diam; lateral stipe extensions (90° to the axis) also present. Conidiogenous apparatus was (40–)56–88(–113) μm long, (45–)67–107(–129) μm wide; primary branches aseptate or 1-septate, (15.7–)18.4–25.9(–30.6) × (3.3–)4–6(–6.5) μm; secondary branches aseptate, (12.7–)14.3–19.6(–22.1) × (3–)3.5–5(–6) μm; tertiary branches aseptate, (9.9–)11.6–15.3(–17.9) × (2.8–)3.6–5.3(–6.4) μm; additional branches (–4), aseptate, (10.3–)11–13.2(–14) × (3–)3.2–4.4(–5) μm; each terminal branch produced 2–4 phialides; phialides doliiform to reniform, hyaline, aseptate, (8–)9.1–11.8(–14) × (2–)2.7–4.1(–6) μm, apex with minute periclinal thickening and inconspicuous collarette. Macroconidia were cylindrical, rounded at both ends, straight, (47–)56–66(–71) × (4–)4.8–5.9(–7) μm (av. = 61 × 5 μm), (1–3) septate, lacking a visible abscission scar, held in parallel cylindrical clusters by colorless slime. Megaconidia and microconidia were not observed.

**Figure 3.**
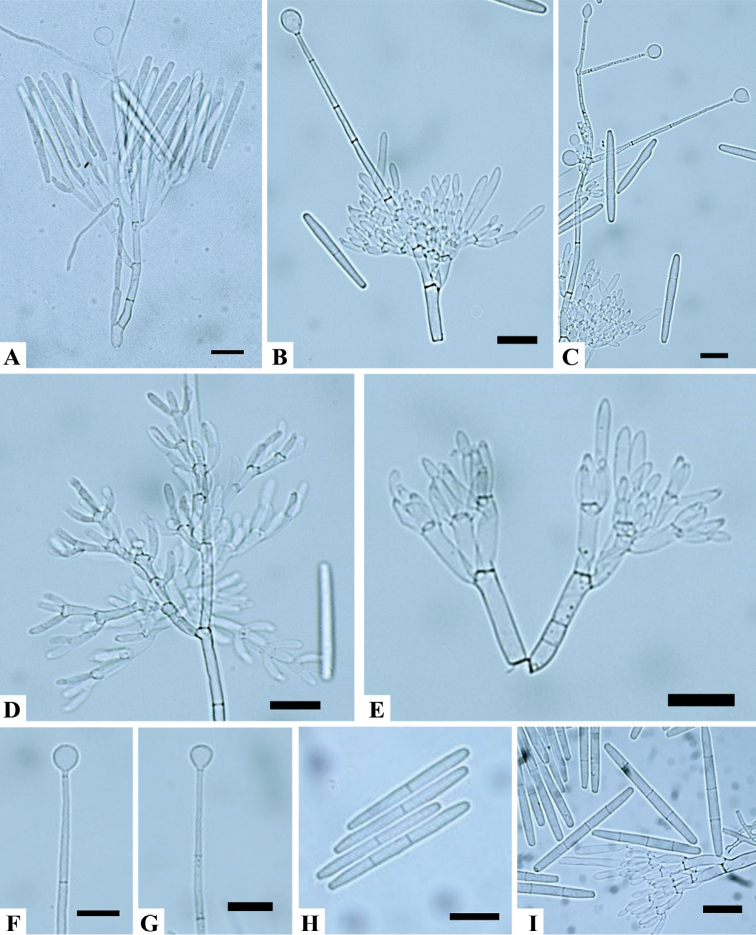
*Calonectriaparagominensis***A, B** macroconidiophore **C** lateral stipe extensions **D, E** conidiogenous apparatus with conidiophore branches and doliiform to reniform phialides **F, G** globose to sphaeropedunculate vesicles **H, I** one, two, and three-septate macroconidia. Scale bars: 20 μm.

##### Culture characteristics.

Colonies formed abundant white aerial mycelium on MEA at 25 °C after seven days, with irregular margins and moderate sporulation. The surface had white to buff outer margins, and sienna to amber in reverse with abundant chlamydospores throughout the medium, forming microsclerotia. The optimal growth temperature was 23.8 °C, with no growth at 5 °C; after seven days, colonies at 10 °C, 15 °C, 20 °C, 25 °C, and 30 °C reached 7 mm, 23 mm, 38.3 mm, 36.1 mm, and 31.8 mm, respectively.

##### Substratum.

Leaves of *E.grandis* × *E.brassiana*.

##### Distribution.

Northeast Brazil.

##### Other specimens examined.

Brazil,• Pará state, Paragominas microregion; From infected leaves of *E.grandis* × *E.brassiana*; 20 Feb. 2020; M.A. Ferreira; cultures PFC2, PFC3, PFC4, PFC5.

##### Notes.

*C.paragominensis* is a new species in the *C.spathiphylli* species complex (Liu et al., 2020). Morphologically, *C.paragominensis* is very similar to *C.densa*, since both form lateral stipe extensions, which have not been reported for the other three species in the complex. However, the macroconidia of *C.paragominensis* (av. 61 × 5 μm) are longer than those of *C.densa* (av. 54 × 6 μm), *C.humicola* (av. 51 × 5 μm) and *C.pseudospathiphylli* (av. 52 × 4 μm) but smaller than those of *C.spathiphylli* (av. 70 × 6 μm).

#### ﻿ Calonectria imperata

Taxon classificationFungiHypocrealesNectriaceae

E.I.Sanchez, T.P.F.Soares & M.A.Ferreira sp. nov.

4843A76F-0B34-56D4-B28F-1E0EEFAC60E7

843461

Fig. 4

##### Etymology.

The term “*imperata*” is in honor of the city of Imperatriz, Brazil, which was close to the place where the fungus was collected.

##### Diagnosis.

*Calonectriaimperata* differs from the phylogenetically closely related species *C.brassiana*, *C.glaebicola*, *C.piauiensis* and *C.venezuelana* with respect to the number of unique alleles and stipe dimensions.

##### Type.

**Brazil**,• Maranhão state, Cidelândia municipality; 5°09'24"S, 47°46'26"W; From infected leaves of *E.urophylla*; 20 Feb. 2020; M.A. Ferreira; **holotype**: UB24350, **ex-type**: CCDCA 11649 = PFC6. GenBank: *act* = ON009351; *cmdA* = OM974330; *his3* = OM974339; *rpb2* = OM974348; *tef1* = OM974357; *tub2* = OM974366.

##### Description.

Sexual morph unknown. Macroconidiophores consisted of a stipe, a suite of penicillate arrangements of fertile branches, a stipe extension, and a terminal vesicle; stipe septate, hyaline, smooth, (135–)151–198(–227) × (2–)2.6–3.4(–4) μm; stipe extension septate, straight to flexuous, (151–)169–220(–254) μm long, (1.5–)1.9–2.7(–3) μm wide at the apical septum, terminating in an ellipsoidal to narrowly obpyriform vesicle (3–)3.1–4.6(–6) μm diam. Conidiogenous apparatus was (50–)66–100(–127) μm long, (41–)62–89(–110) μm wide; primary branches aseptate, (14.6–)19–24.8(–28.5) × (2.5–)3.2–4(–4.5) μm; secondary branches aseptate, (12.1–)13.5–18.2(–24.2) × (2.3–)2.8–3.7(–4) μm; tertiary branches aseptate, (10.1–)11–15(–18.1) × (1.9–)2.3–3.2(–4.1) μm; each terminal branch producing 2–4 phialides; phialides doliiform to reniform, hyaline, aseptate, (8–)9.1–13(–15) × (2–)2.7–3.3(–4) μm, apex with minute periclinal thickening and inconspicuous collarette. Macroconidia were cylindrical, rounded at both ends, straight, (38–)43–49(–52) × (2–)2.7–3.2(–4) μm (av. = 46 × 3 μm), (–1) septate, lacking a visible abscission scar, held in parallel cylindrical clusters by colorless slime. Megaconidia and microconidia were not observed.

**Figure 4.**
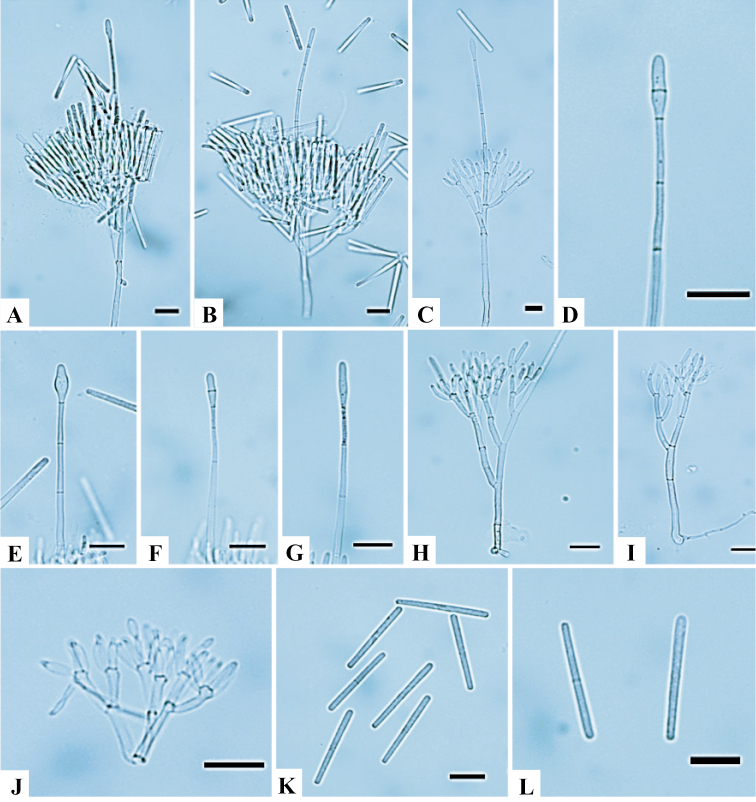
*Calonectriaimperata***A–C** macroconidiophore **D–G** ellipsoidal to narrowly obpyriform vesicles **H–J** conidiogenous apparatus with conidiophore branches and doliiform to reniform phialides **K, L** macroconidia. Scale bars: 20 μm.

##### Culture characteristics.

Colonies formed moderate aerial mycelium on MEA at 25 °C after seven days, with moderate sporulation. The surface had white to buff outer margins, and sepia to umber in reverse with abundant chlamydospores throughout the medium, forming microsclerotia. The optimal growth temperature was 25 °C, with no growth at 5 °C; after seven days, colonies at 10 °C, 15 °C, 20 °C, 25 °C, and 30 °C reached 10.1 mm, 25.5 mm, 29.1 mm, 44.5 mm, and 40.6 mm, respectively.

##### Substratum.

Leaves of *E.urophylla*.

##### Distribution.

Northeast Brazil.

##### Other specimens examined.

Brazil,• Maranhão state, Cidelândia municipality; 5°09'24"S, 47°46'26"W; From infected leaves of *E.urophylla*; 20 Feb. 2020; M.A. Ferreira; cultures PFC7, PFC8, PFC9. Brazil• Maranhão state, Itinga do Maranhão; 4°34'43"S, 47°29'48"W; from infected leaves of *E.urophylla*; 20 Feb. 2020; M.A. Ferreira; culture PFC9.

##### Notes.

*C.imperata* is a new species in the *C.candelabrum* species complex (Liu et al., 2020). Morphologically, *C.imperata* is very similar to its closest relatives, from which it can be distinguished based on stipe dimensions and phylogenetic inference. Stipe of *C.imperata* (135–227 × 2–4 μm) is larger than those of *C.piauiensis* (50–110 × 4–6 μm), *C.glaebicola* (50–130 × 5–7 μm), and *C.venezuelana* (35–100 × 4–8 μm) but narrower than those of *C.brassiana* (55–155 × 5–8 μm). Additionally, *C.imperata* lacks lateral stipe extensions, which are present in *C.piauiensis*.

### ﻿Pathogenicity tests

**Figure 5.**
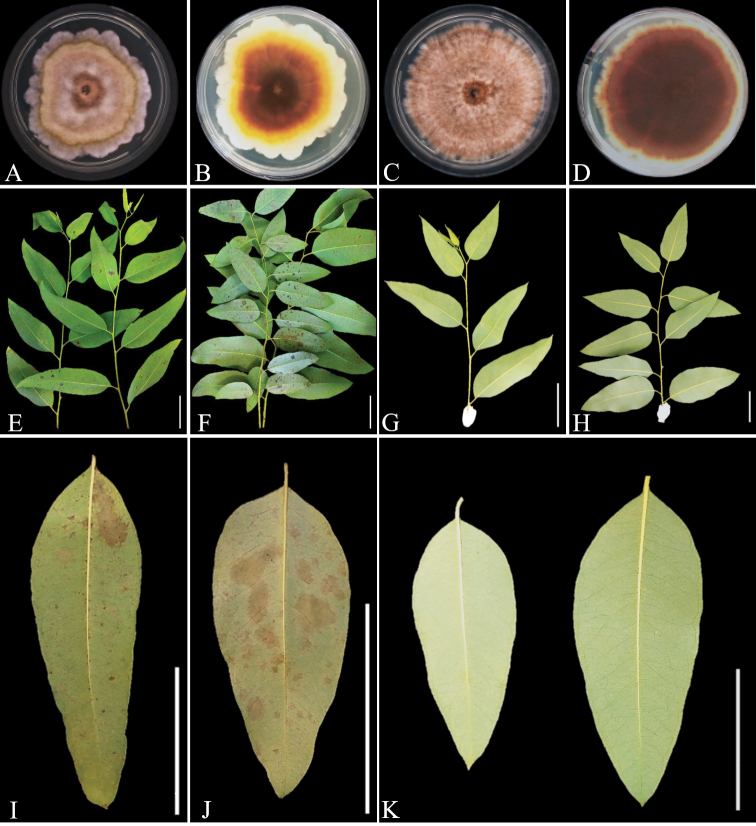
Pathogenicity tests on leaves of *Eucalyptus* genotypes **A, B** surface and reverse of *C.paragominensis* on MEA plates after 14 days grown at 25 °C **C, D** surface and reverse of *C.imperata* on MEA plates after 14 days grown at 25 °C **E, I** lesions on leaves of *E.grandis* × *E.brassiana* induced by *C.paragominensis* 72 h after inoculation **F, J** lesions on leaves of *E.urophylla* induced by *C.imperata* 72 h after inoculation **G, H, K** no disease symptoms on leaves inoculated with sterile water (negative controls). Scale bars: 5 cm (**E–K**).

The conidia suspensions of the representative isolates of *C.paragominensis* and *C.imperata* produced lesion symptoms on leaves (Fig. 5E, F, I, J), but no lesions were observed on the negative control inoculations (Fig. 5G, H, K). The pathogens were reisolated from inoculated leaves but not from the negative controls and identified by the same morphological characteristics as the originally inoculated species, thus, fulfilling the requirements of Koch’s postulates.

## ﻿Discussion

Two new species of *Calonectria* isolated from diseased *Eucalyptus* leaves were identified based on phylogenetic analyses of six gene regions and on morphological comparisons. These two species were named *C.paragominensis* and *C.imperata*.

*Calonectriaparagominensis* is a new species in the *C.spathiphylli* complex. The five species identified and described in *C.spathiphylli* complex are *C.densa*, *C.humicola*, *C.spathiphylli*, *C.pseudospathiphylli*, and *C.paragominensis*, where *C.paragominensis* can be differentiated morphologically with respect to the macroconidia dimensions (El-Gholl et al. 1992; Kang et al. 2001; Crous 2002; Lombard et al. 2010b). These species are characterized by presenting globoid to ovoid to sphaeropedunculate terminal vesicles (Kang et al. 2001; Crous 2002; Lombard et al. 2010b). *Calonectriaspathiphylli* is described as heterothallic (El-Gholl et al. 1992), *C.densa* as putatively heterothallic (Li et al. 2020) and *C.pseudospatiphilly* as homothallic (Kang et al. 2001). The *C.humicola* mating type has not been indicated (Lombard et al. 2010b). Here, *C.paragominensis* is described as putatively homothallic based on PCR amplification of the mating-type genes. Regarding pathogenicity, *C.paragominensis* is pathogenic to *Eucalyptus* sp., *C.spathiphylli* is pathogenic to *Sapthiphyllum* sp. *Heliconia* sp. *Ludwigia* sp. *Strelitzia* sp. and *Eugenia* sp. (El-Gholl et al. 1992; Poltronieri et al. 2011). *Calonectriadensa*, *C.humicola*, and *C.pesudospathiphylli* were isolated from soil, and their pathogenicity has not been indicated (Kang et al. 2001; Lombard et al. 2010b). In addition to *C.paragominensis*, only *C.spathiphylli* has been indicated to be present in Brazil (Reis et al. 2004; Poltronieri et al. 2011).

*Calonectriaimperata* is a new species in the *C.candelabrum* complex. Species in this complex are characterized by presenting ellipsoidal to obpyriform terminal vesicles, in both heterothallic and homothallic species, and occur in Africa, Asia, Europe, North and South America, and Oceania (Liu et al. 2020). Of the 21 species in the *C.candelabrum* complex (Crous et al. 2018, 2019; Liu et al. 2020), 17 have been found in Brazil (Schoch et al. 1999; Crous et al. 2018, 2019; Liu et al. 2020). *Calonectriaimperata* is phylogenetically closely related to *C.brassiana*, *C.glaebicola*, *C.piauiensis* and *C.venezuelana*, which can be differentiated with respect to the number of unique alleles and stipe dimensions (Alfenas et al. 2015; Lombard et al. 2016). *Calonectriabrassiana*, *C.glaebicola*, and *C.piauiensis* were found in Brazil, isolated from soil samples of *Eucalyptus* plantations, but only *C.glaebicola* has been confirmed to be pathogenic to *Eucalyptus* sp. (Alfenas et al. 2015). *Calonectriavenezuelana* was reported in Venezuela, similarly, isolated from soil samples, but its pathogenicity has not been indicated (Lombard et al. 2016). The mating-type for *C.brassiana*, *C.glaebicola*, *C.piauiensis* and *C.venezuelana* has not been determined (Liu et al. 2020).

Pathogenicity tests showed that *C.paragominensis* and *C.imperata* are pathogenic to *E.grandis* × *E.brassiana* hybrid genotype and *E.urophylla* genotype, respectively. Although the death of *Eucalyptus* trees due to CLB is not common, it affects *Eucalyptus* plants most severely from six months to 2–3 years after planting (Graça et al. 2009). Although the economic loss due to defoliation caused by CLB has not been quantified directly, according to artificial pruning studies conducted by Pulrolnik et al. (2005) and Pires (2000), when the loss of branches is equal to or greater than 75% of *E.grandis* seedlings of one year of age, the volumetric productivity has decreased by 45% by the time they reach seven years old. Therefore, it has been inferred that in susceptible clones, the pathogen can cause economic losses, since under favorable conditions, infection by *Calonectria* can result in severe defoliation (Soares et al. 2018). Additionally, Miranda et al. (2021) indicated a potential growth loss of 19.8 to 39.6% due to CLB, and by using the estimates of growth reduction from Pires (2000) as a baseline, concluded that a reduction in the volumetric increment to the order of 39.6% may result in an economic loss of R$ 4291.00 per ha, considering a price of *Eucalyptus* wood as R$ 38.70 per m^3^ (IEA 2020) and a production of 280 m^3^·ha^-1^ in the first 7-year rotation. Therefore, accurate diagnoses of plant diseases and identification of their casual agents are fundamental in promoting the development of effective disease management strategies (Wingfield et al. 2015; Liu and Chen 2017).

In this study, we described two new *Calonectria* species, both isolated from diseased *Eucalyptus* leaves from commercial plantations localized in a tropical zone. These results suggest that there are still more *Calonectria* species to be discovered in Brazil, and that they require careful monitoring, since this knowledge could facilitate the development of resistant *Eucalyptus* clones.

## Supplementary Material

XML Treatment for Calonectria paragominensis

XML Treatment for Calonectria imperata

## Supplementary materials

Supplementary material 1 Figures S1–S6 Data type Phylogenetic trees (pdf file) Explanation note **Figure S1.** Phylogenetic tree based on maximum likelihood analysis of *act* gene region. Bootstrap support values ≥ 80% for maximum parsimony (MP), Ultrafast bootstrap support values ≥ 95% for maximum likelihood (ML), and posterior probability (PP) values ≥ 0.95 from BI analyses are presented at the nodes (MP/ML/PP). Bootstrap values below 80% (MP), 95% (ML) and posterior probabilities below 0.80 are marked with “-”. Ex-type isolates are indicated by “▲”, isolates highlighted in bold were sequenced in this study, and novel species are in blue and orange. *C.gracilipes* was used as outgroup. The scale bar indicates the number of nucleotide substitutions per site. **Figure S2.** Phylogenetic tree based on maximum likelihood analysis of *cmdA* gene region. Bootstrap support values ≥ 80% for maximum parsimony (MP), Ultrafast bootstrap support values ≥ 95% for maximum likelihood (ML), and posterior probability (PP) values ≥ 0.95 from BI analyses are presented at the nodes (MP/ML/PP). Bootstrap values below 80% (MP), 95% (ML) and posterior probabilities below 0.80 are marked with “-”. Ex-type isolates are indicated by “▲”, isolates highlighted in bold were sequenced in this study, and novel species are in blue and orange. *C.gracilipes* was used as outgroup. The scale bar indicates the number of nucleotide substitutions per site. **Figure S3.** Phylogenetic tree based on maximum likelihood analysis of *his3* gene region. Bootstrap support values ≥ 80% for maximum parsimony (MP), Ultrafast bootstrap support values ≥ 95% for maximum likelihood (ML), and posterior probability (PP) values ≥ 0.95 from BI analyses are presented at the nodes (MP/ML/PP). Bootstrap values below 80% (MP), 95% (ML) and posterior probabilities below 0.80 are marked with “-”. Ex-type isolates are indicated by “▲”, isolates highlighted in bold were sequenced in this study, and novel species are in blue and orange. *C.gracilipes* was used as outgroup. The scale bar indicates the number of nucleotide substitutions per site. **Figure S4.** Phylogenetic tree based on maximum likelihood analysis of *rpb2* gene region. Bootstrap support values ≥ 80% for maximum parsimony (MP), Ultrafast bootstrap support values ≥ 95% for maximum likelihood (ML), and posterior probability (PP) values ≥ 0.95 from BI analyses are presented at the nodes (MP/ML/PP). Bootstrap values below 80% (MP), 95% (ML) and posterior probabilities below 0.80 are marked with “-”. Ex-type isolates are indicated by “▲”, isolates highlighted in bold were sequenced in this study, and novel species are in blue and orange. *C.gracilipes* was used as outgroup. The scale bar indicates the number of nucleotide substitutions per site. **Figure S5.** Phylogenetic tree based on maximum likelihood analysis of *tef1* gene region. Bootstrap support values ≥ 80% for maximum parsimony (MP), Ultrafast bootstrap support values ≥ 95% for maximum likelihood (ML), and posterior probability (PP) values ≥ 0.95 from BI analyses are presented at the nodes (MP/ML/PP). Bootstrap values below 80% (MP), 95% (ML) and posterior probabilities below 0.80 are marked with “-”. Ex-type isolates are indicated by “▲”, isolates highlighted in bold were sequenced in this study, and novel species are in blue and orange. *C.gracilipes* was used as outgroup. The scale bar indicates the number of nucleotide substitutions per site. **Figure S6.** Phylogenetic tree based on maximum likelihood analysis of *tub2* gene region. Bootstrap support values ≥ 80% for maximum parsimony (MP), Ultrafast bootstrap support values ≥ 95% for maximum likelihood (ML), and posterior probability (PP) values ≥ 0.95 from BI analyses are presented at the nodes (MP/ML/PP). Bootstrap values below 80% (MP), 95% (ML) and posterior probabilities below 0.80 are marked with “-”. Ex-type isolates are indicated by “▲”, isolates highlighted in bold were sequenced in this study, and novel species are in blue and orange. *C.gracilipes* was used as outgroup. The scale bar indicates the number of nucleotide substitutions per site. https://binary.pensoft.net/file/721461This dataset is made available under the Open Database License (http://opendatacommons.org/licenses/odbl/1.0/). The Open Database License (ODbL) is a license agreement intended to allow users to freely share, modify, and use this Dataset while maintaining this same freedom for others, provided that the original source and author(s) are credited.Enrique I. Sanchez-Gonzalez, Thaissa de Paula Farias Soares, Talyta Galafassi Zarpelon, Edival Angelo Valverde Zauza, Reginaldo Gonçalves Mafia, Maria Alves Ferreira
