# Supplementary material for: ﻿Two new species of Calonectria (Hypocreales, Nectriaceae) causing Eucalyptus leaf blight in Brazil
Source: MycoKeys. 2022 Jul 29;91:169–97. doi: 10.3897/mycokeys.91.84896 (PMC9849068; doi:10.3897/mycokeys.91.84896)
Supplement: Supplementary material 1 — Figures S1–S6 [file mycokeys-91-169-s001.pdf]

Supplementary material 1

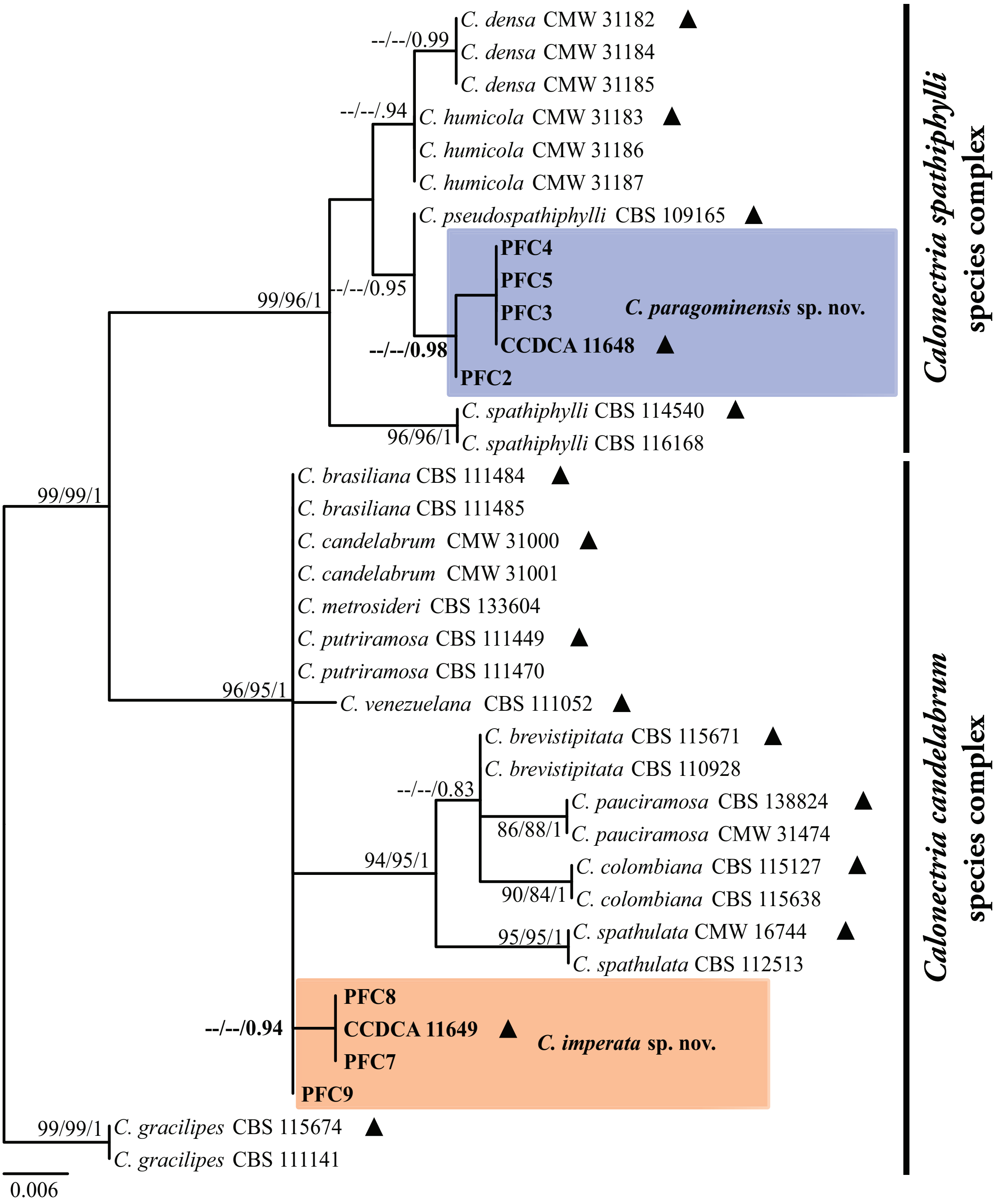

Figure S1. Phylogenetic tree based on maximum likelihood analysis of *act* gene region. Bootstrap support values ≥ 80% for maximum parsimony (MP), Ultrafast bootstrap support values ≥ 95% for maximum likelihood (ML), and posterior probability (PP) values ≥ 0.95 from BI analyses are presented at the nodes (MP/ML/PP). Bootstrap values below 80% (MP), 95% (ML) and posterior probabilities below 0.80 are marked with "-". Ex-type isolates are indicated by "▲", isolates highlighted in bold were sequenced in this study, and novel species are in blue and orange. *C. gracilipes* was used as outgroup. The scale bar indicates the number of nucleotide substitutions per site.

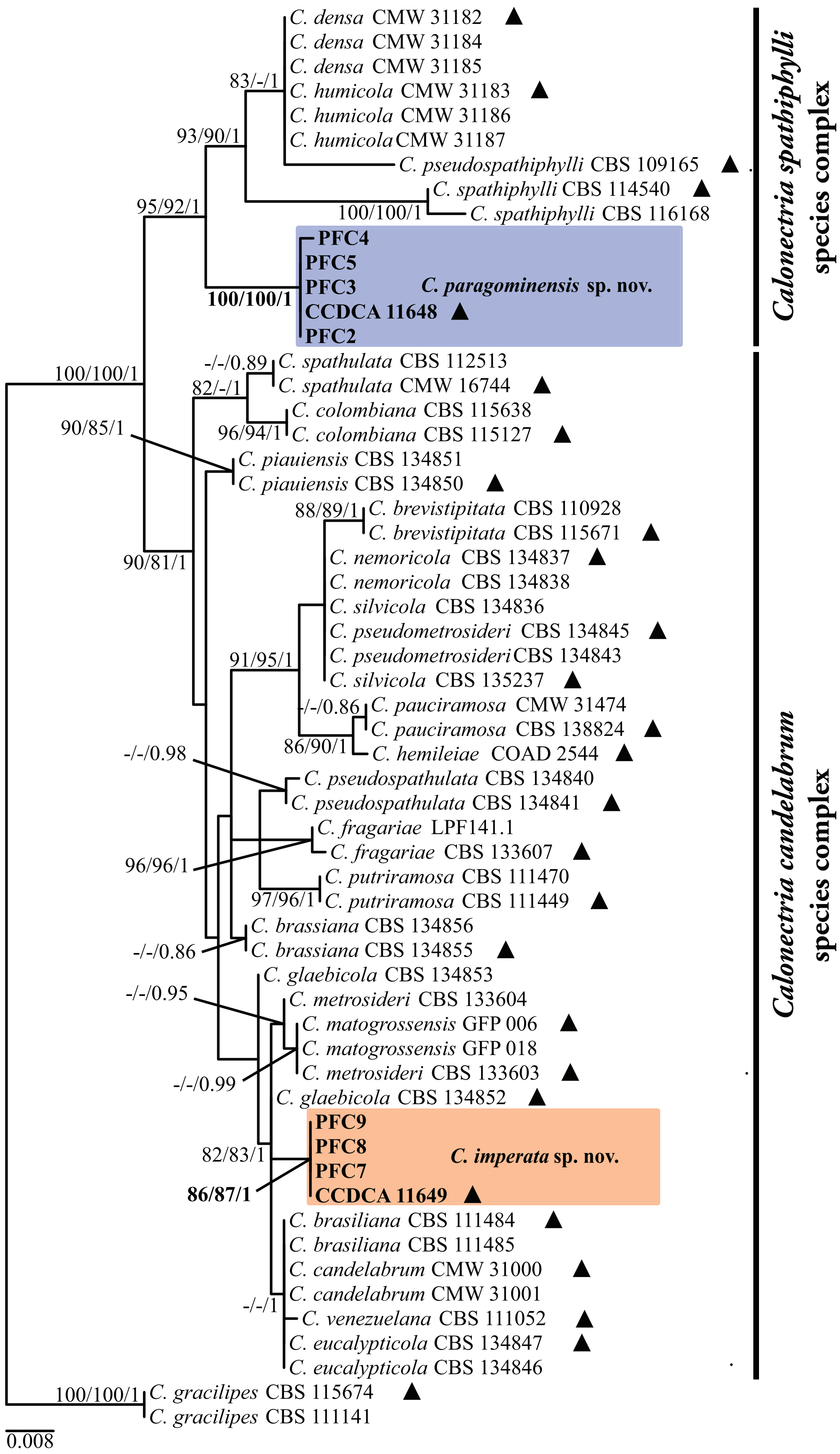

Figure S2. Phylogenetic tree based on maximum likelihood analysis of *cmdA* gene region. Bootstrap support values ≥ 80% for maximum parsimony (MP), Ultrafast bootstrap support values ≥ 95% for maximum likelihood (ML), and posterior probability (PP) values ≥ 0.95 from BI analyses are presented at the nodes (MP/ML/PP). Bootstrap values below 80% (MP), 95% (ML) and posterior probabilities below 0.80 are marked with "-". Ex-type isolates are indicated by "▲", isolates highlighted in bold were sequenced in this study, and novel species are in blue and orange. *C. gracilipes* was used as outgroup. The scale bar indicates the number of nucleotide substitutions per site.

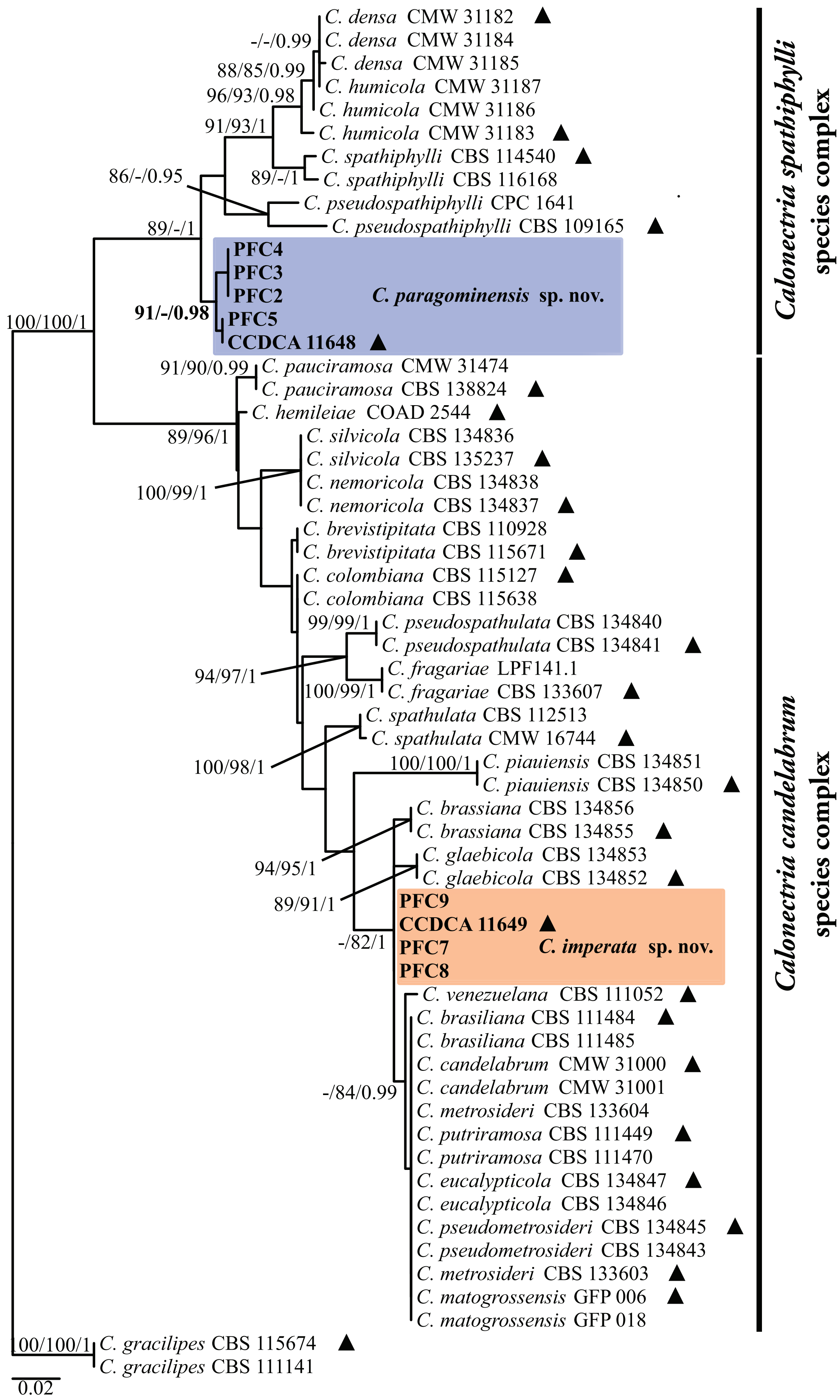

Figure S3. Phylogenetic tree based on maximum likelihood analysis of *his3* gene region. Bootstrap support values ≥ 80% for maximum parsimony (MP), Ultrafast bootstrap support values ≥ 95% for maximum likelihood (ML), and posterior probability (PP) values ≥ 0.95 from BI analyses are presented at the nodes (MP/ML/PP). Bootstrap values below 80% (MP), 95% (ML) and posterior probabilities below 0.80 are marked with "-". Ex-type isolates are indicated by "▲", isolates highlighted in bold were sequenced in this study, and novel species are in blue and orange. *C. gracilipes* was used as outgroup. The scale bar indicates the number of nucleotide substitutions per site.

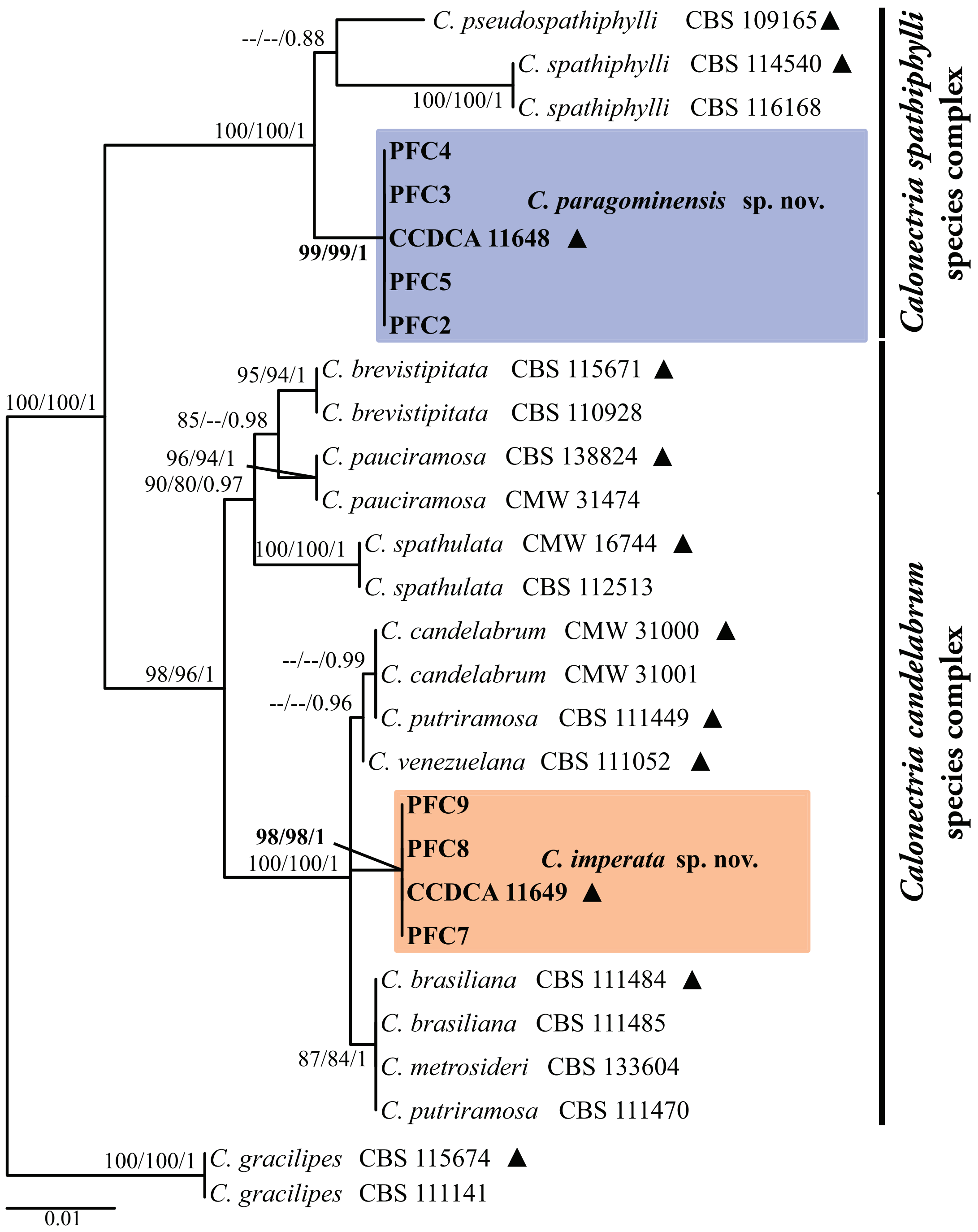

Figure S4. Phylogenetic tree based on maximum likelihood analysis of *rpb2* gene region. Bootstrap support values ≥ 80% for maximum parsimony (MP), Ultrafast bootstrap support values ≥ 95% for maximum likelihood (ML), and posterior probability (PP) values ≥ 0.95 from BI analyses are presented at the nodes (MP/ML/PP). Bootstrap values below 80% (MP), 95% (ML) and posterior probabilities below 0.80 are marked with "-". Ex-type isolates are indicated by "▲", isolates highlighted in bold were sequenced in this study, and novel species are in blue and orange. *C. gracilipes* was used as outgroup. The scale bar indicates the number of nucleotide substitutions per site.

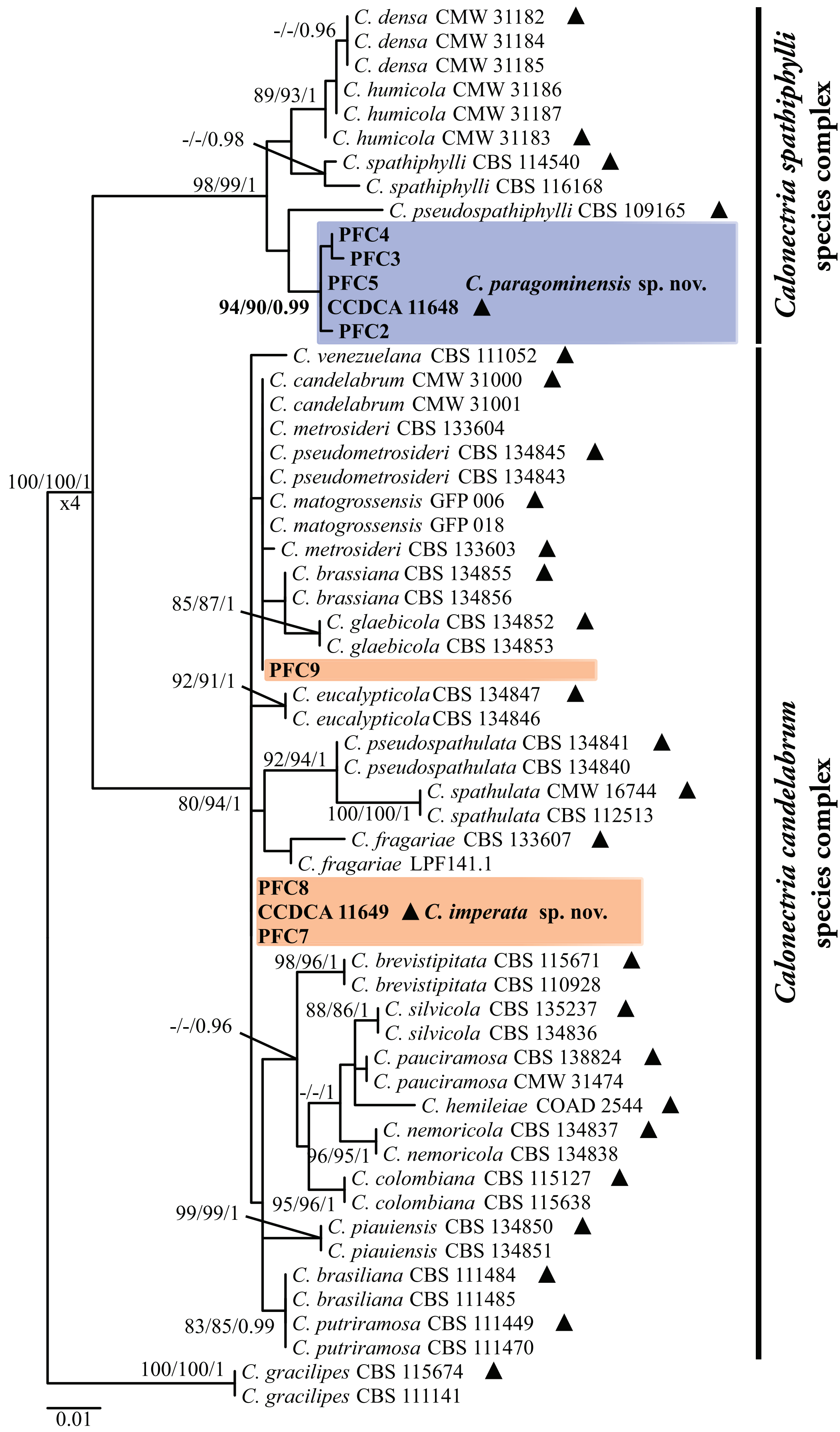

Figure S5. Phylogenetic tree based on maximum likelihood analysis of *tef1* gene region. Bootstrap support values ≥ 80% for maximum parsimony (MP), Ultrafast bootstrap support values ≥ 95% for maximum likelihood (ML), and posterior probability (PP) values ≥ 0.95 from BI analyses are presented at the nodes (MP/ML/PP). Bootstrap values below 80% (MP), 95% (ML) and posterior probabilities below 0.80 are marked with "-". Ex-type isolates are indicated by "▲", isolates highlighted in bold were sequenced in this study, and novel species are in blue and orange. *C. gracilipes* was used as outgroup. The scale bar indicates the number of nucleotide substitutions per site.

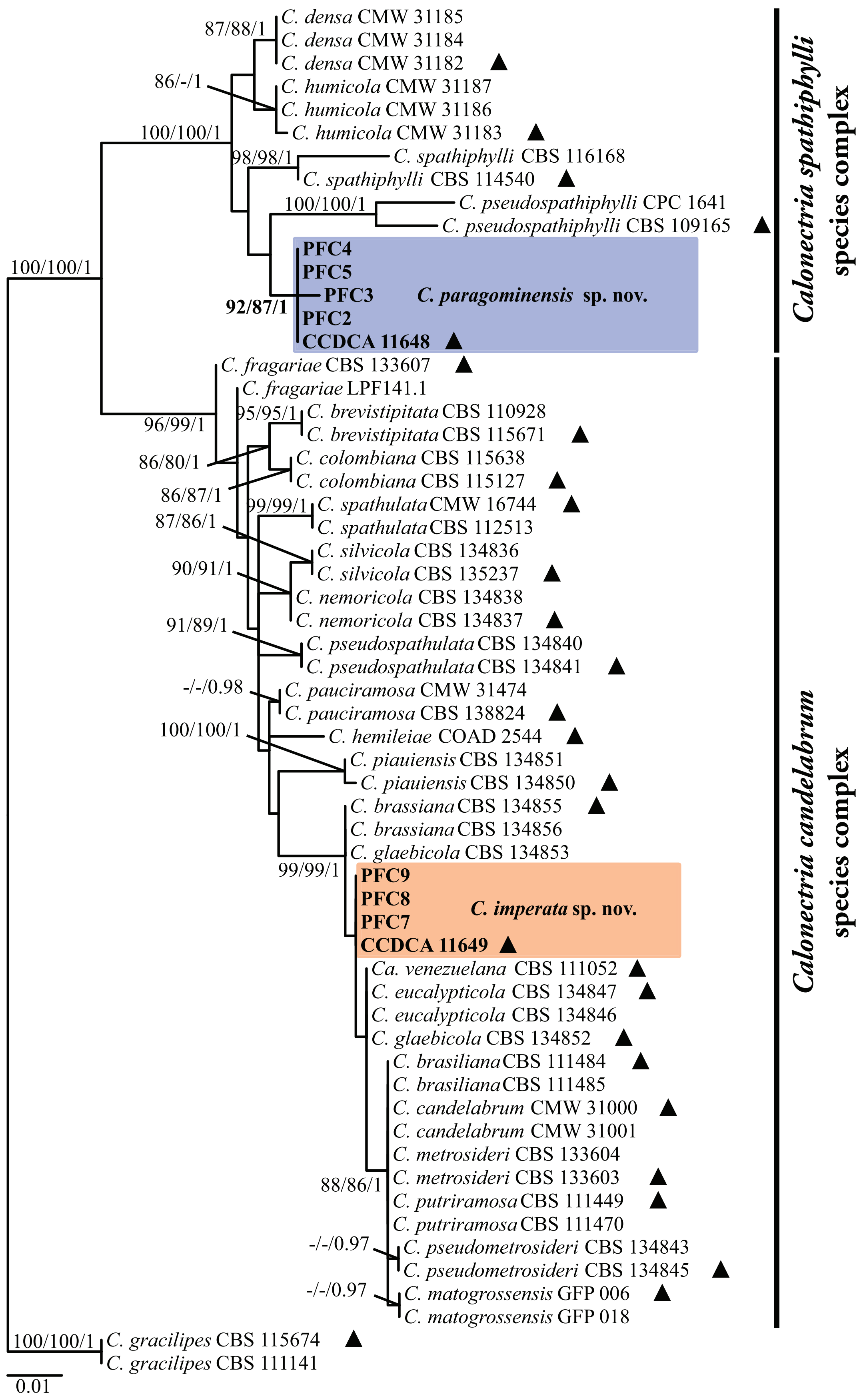

Figure S6. Phylogenetic tree based on maximum likelihood analysis of *tub2* gene region. Bootstrap support values ≥ 80% for maximum parsimony (MP), Ultrafast bootstrap support values ≥ 95% for maximum likelihood (ML), and posterior probability (PP) values ≥ 0.95 from BI analyses are presented at the nodes (MP/ML/PP). Bootstrap values below 80% (MP), 95% (ML) and posterior probabilities below 0.80 are marked with "-". Ex-type isolates are indicated by "▲", isolates highlighted in bold were sequenced in this study, and novel species are in blue and orange. *C. gracilipes* was used as outgroup. The scale bar indicates the number of nucleotide substitutions per site.
